# Supplementary material for: Large organized chromatin lysine domains help distinguish primitive from differentiated cell populations
Source: Nat Commun. 2021 Jan 21;12:499. doi: 10.1038/s41467-020-20830-9 (PMC7820432; doi:10.1038/s41467-020-20830-9)
Supplement: Supplementary file 2 — Description of Additional Supplementary Files [file 41467_2020_20830_MOESM2_ESM.pdf]

Description of Additional Supplementary Files

File Name: Supplementary Software

Description: A copy of all the call peak files used to generate figures of the manuscript.
